# Conscious Multisensory Integration: Introducing a Universal Contextual Field in Biological and Deep Artificial Neural Networks

**DOI:** 10.3389/fncom.2020.00015

**Published:** 2020-05-19

**Authors:** Ahsan Adeel

**Affiliations:** ^1^Oxford Computational Neuroscience, Nuffield Department of Surgical Sciences, John Radcliffe Hospital, University of Oxford, Oxford, United Kingdom; ^2^School of Mathematics and Computer Science, University of Wolverhampton, Wolverhampton, United Kingdom

**Keywords:** universal contextual field, pyramidal cell, multisensory integration, coherent infomax neuron, contextually-adaptive neuron, deep neural network, audio-visual speech processing

## Abstract

Conscious awareness plays a major role in human cognition and adaptive behavior, though its function in multisensory integration is not yet fully understood, hence, questions remain: How does the brain integrate the incoming multisensory signals with respect to different external environments? How are the roles of these multisensory signals defined to adhere to the anticipated behavioral-constraint of the environment? This work seeks to articulate a novel theory on conscious multisensory integration (CMI) that addresses the aforementioned research challenges. Specifically, the well-established contextual field (CF) in pyramidal cells and coherent infomax theory (Kay et al., 1998; Kay and Phillips, 2011) is split into two functionally distinctive integrated input fields: local contextual field (LCF) and universal contextual field (UCF). LCF defines the modulatory sensory signal coming from some other parts of the brain (in principle from anywhere in space-time) and UCF defines the outside environment and anticipated behavior (based on past learning and reasoning). Both LCF and UCF are integrated with the receptive field (RF) to develop a new class of contextually-adaptive neuron (CAN), which adapts to changing environments. The proposed theory is evaluated using human contextual audio-visual (AV) speech modeling. Simulation results provide new insights into contextual modulation and selective multisensory information amplification/suppression. The central hypothesis reviewed here suggests that the pyramidal cell, in addition to the classical excitatory and inhibitory signals, receives LCF and UCF inputs. The UCF (as a steering force or tuner) plays a decisive role in precisely selecting whether to amplify/suppress the transmission of relevant/irrelevant feedforward signals, without changing the content e.g., which information is worth paying more attention to? This, as opposed to, unconditional excitatory and inhibitory activity in existing deep neural networks (DNNs), is called conditional amplification/suppression.

## 1. Introduction

What is conscious awareness? Think of a well-trained and experienced car driver who automatically identifies and follows the traffic protocols in different surrounding environments (e.g., street, highway, city centre) by simply interpreting the visual scenes directly (such as buildings, school etc.). Similarly, imagine a car with slightly defective parking sensors that sometimes miscalculates the distance to the nearest object. In this case, the audio input is ambiguous and the driver cannot fully rely on parking sensors for precise maneuvering decisions, e.g., while reversing the car. To tackle this problem, the driver automatically starts utilizing visual cues to leverage the complementary strengths of both ambiguous sound (reversing-beeps) and visuals for optimized decision making. These are a few examples of conscious awareness, where the external environment helps establishing the anticipated behavior and the corresponding optimal roles of incoming multisensory signals.

Nonetheless, it raises crucial questions: How does it happen in the brain? How do the incoming sensory signals (such as vision and sound) integrate with respect to the situation? How does a neuron originate a precise control command complying with the anticipated behavioral-constraint of the environment? Certainly, defining the context and its relevant features knowing when a change in context has taken place are challenging problems in modeling human behavior (Gonzalez et al., 2008). It is also claimed in the literature that context could be of infinite dimensions but humans have a unique capability of correlating the significant context and set its boundaries intuitively (Gonzalez et al., 2008). However, once the context is identified, it is relatively easy to utilize and set its bounds to more precisely define the search space for the selection of best possible decision (Gonzalez et al., 2008).

**Figure 1:**
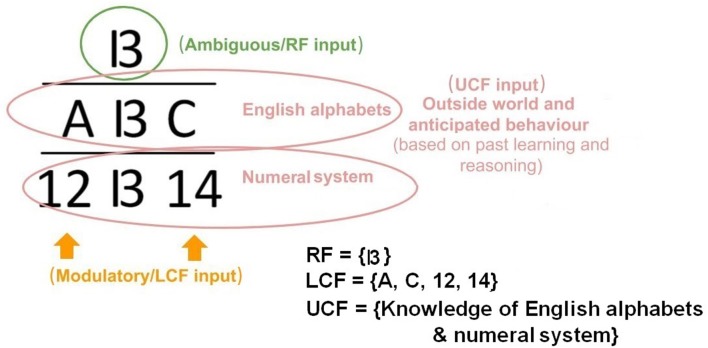
Ambiguous decision making and contextual modulation.

**Figure 2:**
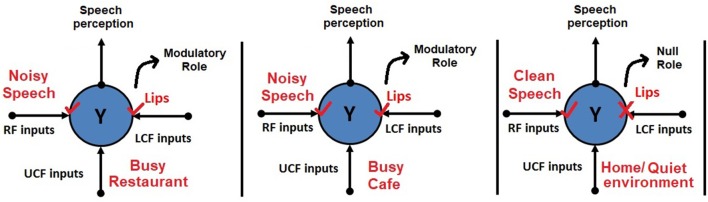
Human AV speech modeling in three different environments. Please note that the role of LCF changes with respect to the outside environment (UCF). For example, in the first two environments, LCF has a modulatory role, whereas in the third environment, it has a Null role.

A simple example of contextual modulation is shown in Figure 1. It can be seen that the ambiguous RF input (in the top row) is interpreted as “B” or “13” depending on the LCF (i.e., “A,” “C,” “12,” and “14”) and UCF (i.e., knowledge of English alphabets and numeral system). Similarly, it is observed that in noisy environments (e.g., a busy restaurant, bar, cocktail party), human brain naturally utilizes other modalities (such as lips, body language, facial expressions) to perceive speech or the conveyed message [i.e., speech-in-noise (SIN) perception] (Sumby and Pollack, 1954; McGurk and MacDonald, 1976; Summerfield, 1979; Patterson and Werker, 2003). This multimodal nature of speech is well-established in the literature; it is understood how speech is produced by the vibration of vocal folds and configuration of the articulatory organs. The developed AV speech processing models are depicted in Figure 2. Two distinctive input variables RF and LCF are defining the incoming sensory inputs (i.e., sound and vision), whereas the UCF input is defining three different surrounding environments: Restaurant, Cafe, and Home. In any environment, multisensory information streams are available, but their optimal integration depends on the outside environment. For example, in a busy cafe and restaurant environment (multi-talker speech perception), the processor utilizes other modalities (i.e., lips as LCF) to disambiguate the noisy speech, whereas in the Home scenario (with little or zero noise), LCF has a Null role.

Hence, coordination and specialization are necessary to produce coherent thoughts, percepts, and actions, which are well-adapted to different situations and long-term goals (Phillips et al., 2015). However, the understanding of specialization and coordination is still a major issue within the cognitive and neurosciences. The hypothesis reviewed in Phillips et al. (2015) suggests that this is mostly achieved by a widely distributed process of contextual modulation, which amplifies and suppresses the transmission of signals that are relevant and irrelevant to current circumstances, respectively. Nevertheless, selective modulation (amplification/attenuation) of incoming multisensory information with respect to the outside world is poorly understood. In addition, not much progress has been made on the use of conscious awareness and contextual modulation to show enhanced processing, learning, and reasoning. In this research article, the aforementioned interesting observations are discussed and a new perspective in terms of CMI with some future research directions is comprehensively presented. The rest of the paper is organized as follows: section 2 discusses the conceptual foundation and motivation that leads to the development of a CAN model. Section 3 presents the CAN and contextually-adaptive neural network (CANN) structures. In sections 4 and 5, the proposed theory is utilized for AV speech processing. Finally, conclusion and future research directions are presented in sections 6 and 7, respectively.

## 2. Motivation and Contribution

For a long time, it was believed that the consciousness depends on neurons firing and synchronization at certain frequency bands. Massimini et al. (2005) suggested that consciousness is not critically dependent on them, but rather on the ability of the brain to integrate multisensory information. This brain ability depends on the effective connectivity^1^ among functionally specialized regions of the thalamocortical system. At a granular level, evidence gathered in the literature suggests that the multisensory interaction emerges at the primary cortical level (Stein and Stanford, 2008; Stein et al., 2009). The divisive/multiplicative gain modulations are widely spread in mammalian neocortex with an indication of amplification or attenuation via contextual modulation (Galletti and Battaglini, 1989; Salinas and Sejnowski, 2001; Phillips et al., 2018).

Scientists have presented several models and empirical results on the role of contextual modulation to disambiguate the ambiguous input (Kay and Phillips, 1997; Kay et al., 1998; Phillips, 2001; Phillips and Silverstein, 2013). For example, in Kay et al. (1998), the contextual modulation was demonstrated using a simple edge detection problem to reveal its effectiveness in recognizing specific patterns with noisy RF input. It was shown how surrounding regions (CF) in different parallel streams helped detecting the edge within any particular region and played a significant role in combating noisy input. This idea is called a coherent infomax theory or coherent infomax neuron (Kay et al., 2017; Lizier et al., 2018).

The physiological studies in Phillips and Singer (1997) have suggested that biological neurons, in addition to the classical excitatory and inhibitory signals, do receive contextual inputs. These contextual inputs possibly fulfill the gain-controlling RC role (Fox and Daw, 1992). The authors in Kepecs and Raghavachari (2002) used a two-compartment model of pyramidal neurons to capture the spatial extent of neuronal morphology. Their study simulated three neurons, each receiving the same α-amino-3-hydroxy-5-methyl-4-isoxazolepropionic acid (AMPA), representing the informational input i.e., a word “green.” The three neurons also received distinct contextual input via NMDA receptors, representing specific noun groups: objects, people and fruits. It is to be noted that the word “green,” when expressed with a contextual input, varies in meaning, e.g., a color green or an unripe fruit. The simulation results showed that even though each neuron received the same strong AMPA input, their firing was uncorrelated and context-dependent.

**Figure 3:**
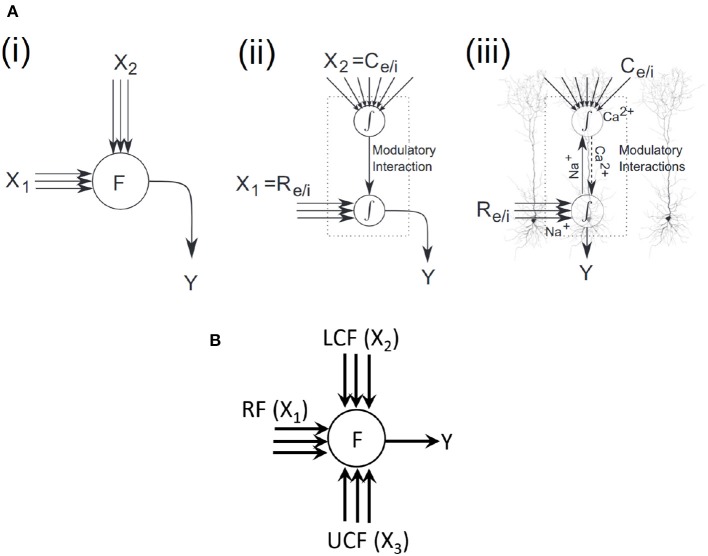
Coherent infomax vs. contextually-adaptive neural processor. **(A)** Coherent infomax neural processor (Phillips et al., 2015; Wibral et al., 2017): (i) with multidimensional inputs *X1;X2*, and output *Y*. (ii) with local weighted summation of inputs: X1 receptive field [excitatory (*R*_*e*_) or inhibitory (*R*_*i*_)] and X2 contextual field [excitatory (*C*_*e*_) or inhibitory (*C*_*i*_)]. (iii) overlay on a layer 5 pyramidal cells. **(B)** Proposed CAN.

An overlay of a coherent infomax neural processor on layer 5 pyramidal cells is shown in Figure 3A (Wibral et al., 2017), highlighting potential parallels to existing physiological mechanisms. In two sites of integration, one is at the soma and the other at the top of the apical trunk. The driving excitatory (*R*_*e*_) or inhibitory (*R*_*i*_) signals arrive via basal and perisomatic synapses, whereas the modulatory excitatory (*C*_*e*_) or inhibitory (*C*_*i*_) signals arrive via synapses on the tuft dendrites at the top of the apical trunk. *Na*^+^ at the somatic integration site initiates sodium spikes that backpropagate up to the apical trunk. *Ca*^2+^ at the apical integration site initiates calcium spikes, which amplify the neural response (Phillips et al., 2015).

In light of the aforementioned literature, in this paper, the contextual AV speech processing is used to demonstrate contextual modulation. Specifically, the CF in coherent infomax theory (Kay et al., 1998; Kay and Phillips, 2011) is split into two fields: LCF and UCF. LCF defines the modulatory sensory signal coming from some other parts of the brain (in principle from anywhere in space-time) and UCF defines the outside environment and anticipated behavior (based on past learning and reasoning). Both LCF and UCF are integrated with the RF to develop a new class of contextually-adaptive neuron, which adapts to changing situations (shown in Figure 3B). For evaluation and comparative analysis, two distinctive multimodal multistreams (lip movements as LCF and noisy speech as RF) are used to study the role of LCF in SIN perception (ranging from a very noisy environment to almost zero noise). Furthermore, going beyond the theory of coherent infomax, UCF is introduced as a fourth new dimension to represent the outside environment and anticipated behavior. Its effectiveness is shown in terms of enhanced learning and processing, using three distinctive multimodal multistreams (lip movements as LCF, noisy speech as RF, and outside environment/anticipated behavior as UCF).

## 3. Contextually-Adaptive Neuron (CAN)

**Figure 4:**
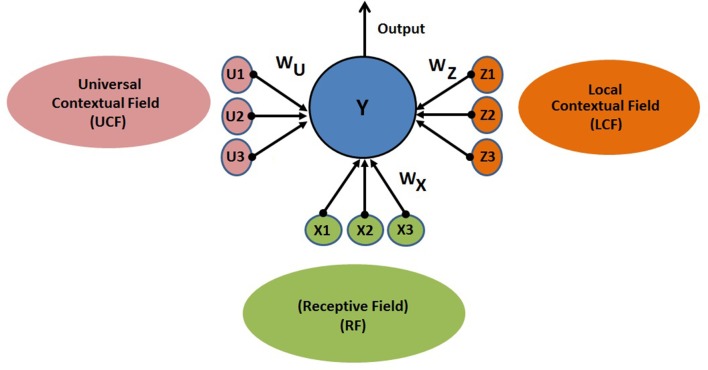
CAN structure: the output depends on three functionally distinctive integrated input variables: driving (RF), modulatory (LCF), and UCF. The RF is defining the ambiguous sensory signal, LCF is defining the modulatory sensory signal coming from other parts of the brain, and UCF is defining the outside world and anticipated behavior. *W*_*X*_, *W*_*Z*_, and *W*_*U*_ are representing the receptive, local contextual, and universal contextual field connections, respectively.

**Figure 5:**
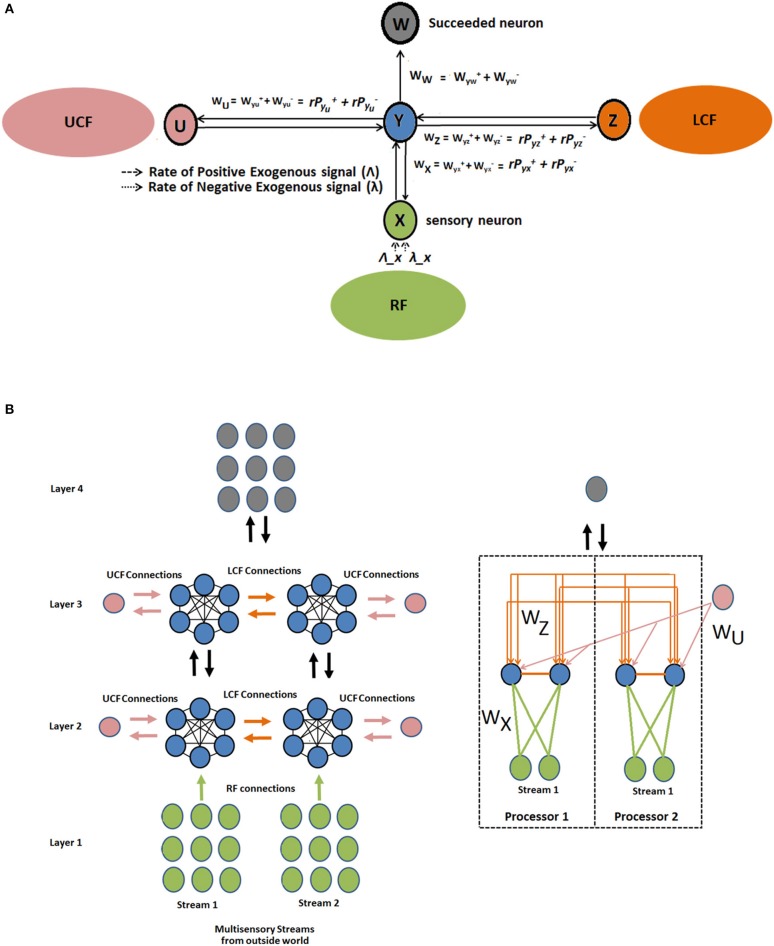
Interaction among RF, LCF, and UCF in CAN and CANN. **(A)** CAN: The filtering rules (precise information integration) are enforced by the positive and negative synaptic weights associated with each input field. **(B)** CANN: multilayered multiunit network of similar CANs, where the CAN in one stream is connected to all other CANs in neighboring streams of the same layer. The figure on the right is providing detailed information about connections using two RF and LCF units in each processor, with one UCF and one output unit each.

The proposed CAN is presented in Figure 4. The output of the neuron depends on three functionally distinctive integrated input variables: driving (RF), modulatory (LCF), and UCF. The RF is defining the ambiguous sensory signal, LCF is defining the modulatory sensory signal coming from other parts of the brain, and UCF is defining the outside world and anticipated behavior. The interaction among RF, LCF, and UCF is shown in Figure 5A. The output is denoted by the random variable Y, whereas X, Z, and U represent RF, LCF, and UCF, respectively. In CANN, the CAN in one stream is connected to all other CANs in the neighboring stream of the same layer as shown in Figure 5B. This is achieved through shared connections among the neurons that guide learning and processing with respect to local and universal contexts.

### 3.1. Mathematical Modeling

The CAN (*Y*) in CANN interacts by exchanging the excitatory and inhibitory spikes probabilistically (in the form of bipolar signal trains). In steady state, the stochastic spiking behavior of the network has a “product form” property (product of firing rates and transition probabilities) which defines a state probability distribution with easily solvable non-linear network equations. The firing from neuron *y* to succeeding neuron *w* in the network is according to the Poisson process, represented by the synaptic weights $w_{yw}^{+}$ = $r_{y}\left[P_{yx}^{+}+P_{yz}^{+}+P_{yu}^{+}\right]$ and $w_{yw}^{-}$ = $r_{y}\left[P_{yx}^{-}+P_{yz}^{-}+P_{yu}^{-}\right]$, where $P_{yx}^{+},P_{yz}^{+},P_{yu}^{+}$ and $P_{yx}^{-},P_{yz}^{-},P_{yu}^{-}$ represent the probabilities of excitatory and inhibitory RF, LCF, and UCF signals, respectively. The term *r*_*y*_ represents the firing rate of the CAN. The terms $w_{yx}^{+}$, $w_{yz}^{+}$, $w_{yu}^{+}$ and $w_{yx}^{-}$, $w_{yz}^{-}$, $w_{yu}^{-}$ represent the RF, LCF, and UCF synaptic weights (i.e., the rates of positive and negative signal transmission) that network learns through the process of learning or training. In the network, CAN receives exogenous signals positive/negative from the inside (within the network) or outside world, according to Poisson arrival streams of rates Λ_*x*_, λ_*x*_, respectively. The potential (*Y*) of the CAN represents its state that increases/decreases with respect to an incoming signal coming from the inside or outside world. The proposed neural structure is implemented using G-networks that possess a product-form asymptotic solution (Gelenbe, 1993a).

The CAN in firing state transmits an impulse to neuron *w* with a Poisson rate (*r*_*y*_) and probability *P*^+^(*y, w*) or *P*^−^(*y, w*) depending on the incoming signal being excitatory or inhibitory. The transmitted signal can also leave the network and go outside the world with probability *d*(*y*) such that:

(1) $$\begin{array}{l} d\left(y\right)+\sum_{x=1}^{N}\left[P^{+}\left(y,x\right)+P^{-}\left(y,x\right)\right]+\sum_{z=1}^{N}\left[P^{+}\left(y,z\right)\right]\\ \;+P^{-}\left(y,z\right)+\sum_{u=1}^{N}\left[P^{+}\left(y,u\right)+P^{-}\left(y,u\right)\right]=1 \end{array}$$

Where,

(2) $$\begin{array}{l} w^{+}\left(y,w\right)=r_{y}\left[P^{+}\left(y,x\right)+P^{+}\left(y,z\right)+P^{+}\left(y,u\right)\right]\geq 0,\\ \;w^{-}\left(y,w\right)=r_{y}\left[P^{-}\left(y,x\right)+P^{-}\left(y,z\right)+P^{-}\left(y,u\right)\right]\geq 0 \end{array}$$

The firing rate of CAN can be written as:

(3) $$\begin{array}{l} r\left(y\right)={\left(1-d\left(y\right)\right)}^{-1}(\sum_{x=1}^{N}\left[w^{+}\left(y,x\right)+w^{-}\left(y,x\right)\right]\\ +\sum_{z=1}^{N}\left[w^{+}\left(y,z\right)+w^{-}\left(y,z\right)\right]+\sum_{u=1}^{N}\left[w^{+}\left(y,u\right)+w^{-}\left(y,u\right)\right]) \end{array}$$

If *Y*(*t*) is the potential of CAN then in *n* number of neurons, vector $\bar{Y\left(t\right)}$ = (*y*_1_(*t*), *y*_2_(*t*), …, *y*_*n*_(*t*)) can be modeled as a continuous-time Markov process. The stationary joint probability of the network is given as:

(4) $$\begin{array}{l} \underset{n\to \infty }{\lim}P\left(\bar{Y\left(t\right)}\right)=y_{1}\left(t\right),y_{2}\left(t\right),\ldots ,y_{n}\left(t\right)=\overset{n}{\underset{y=1}{\prod }}\left(1-q_{y}\right)q_{y}^{ny},\\ q_{y}=\frac{Q_{Y}^{+}}{r_{y}+Q_{Y}^{-}} \end{array}$$

where $Q_{Y}^{+}$ and $Q_{Y}^{-}$ are the average rates of +ive and -ive signals at the CAN (*y*), given as:

(5) $$\begin{array}{l} Q_{Y}^{+}=\overset{N}{\underset{x=1}{\sum }}q_{x}w^{+}\left(y,x\right)+\overset{N}{\underset{z=1}{\sum }}q_{z}w^{+}\left(y,z\right)+\overset{N}{\underset{u=1}{\sum }}q_{u}w^{+}\left(y,u\right) \end{array}$$

(6) $$\begin{array}{l} Q_{Y}^{-}=\overset{N}{\underset{x=1}{\sum }}q_{x}w^{-}\left(y,x\right)+\overset{N}{\underset{z=1}{\sum }}q_{z}w^{-}\left(y,z\right)+\overset{N}{\underset{u=1}{\sum }}q_{u}w^{-}\left(y,u\right) \end{array}$$

The probability that CAN (*Y*) is excited can be written as:

(7) $$\begin{array}{l} q_{y}=\frac{\sum_{x=1}^{N}q_{x}w^{+}\left(y,x\right)+\sum_{z=1}^{N}q_{z}w^{+}\left(y,z\right)+\sum_{u=1}^{N}q_{u}w^{+}\left(y,u\right)}{\left[W_{W}^{+}+W_{W}^{-}\right]+\sum_{w=1}^{N}q_{x}w^{-}\left(y,x\right)+\sum_{z=1}^{N}q_{z}w^{-}\left(y,z\right)+\sum_{u=1}^{N}q_{u}w^{-}\left(y,u\right)} \end{array}$$

where *w*^+^(*y, x*), *w*^−^(*y, x*), *w*^+^(*y, z*), *w*^−^(*y, z*) *w*^+^(*y, u*), *w*^+^(*y, u*) are the positive and negative RF, LCF, and UCF weights. $W_{W}^{+}$ and $W_{W}^{-}$ are the positive and negative weights between CAN and succeeded neuron *w*. For training and weights update, state-of-the-art gradient descent algorithm is used (Gelenbe, 1993b). The RF input (*q*_*x*_) is given as:

(8) $$\begin{array}{l} q_{x}=\frac{Q_{x}^{+}}{\left[w{\left(x,y\right)}^{+}+w{\left(x,y\right)}^{-}\right]+Q_{x}^{-}} \end{array}$$

(9) $$\begin{array}{l} Q_{x}^{+}=\Lambda_{x}+\overset{N}{\underset{v=1}{\sum }}q_{v}w^{+}\left(x,v\right) \end{array}$$

(10) $$\begin{array}{l} Q_{x}^{-}=\lambda_{x}+\overset{N}{\underset{v=1}{\sum }}q_{v}w^{-}\left(x,v\right) \end{array}$$

where *q*_*v*_ is the potential of the preceding neuron *v* and *q*_*u*_ and *q*_*z*_ are potentials of the incoming UCF and LCF neurons, respectively. It is to be noted that *w*(*x, y*)^+^ and *w*(*y, x*)^+^ are different.

### 3.2. Information Decomposition

**Figure 6:**
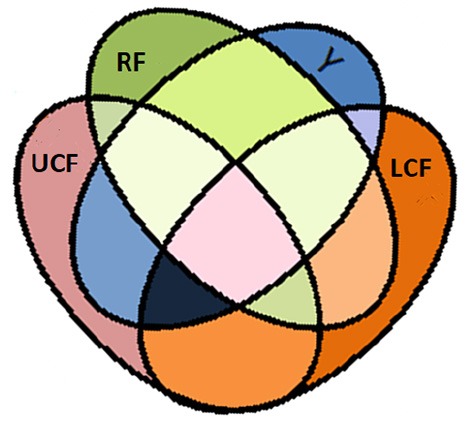
Venn diagram of information theoretic measures for distinctive integrated input variables RF, LCF, and UCF represented by the green ellipse, orange ellipse, and grayish pink ellipse, respectively. The output (Y) is represent by the blue ellipse. The UCF (U) and associated *H*(*U*|*X, Y, Z*) is interpreted as the information contained in U but not in X, Y, and Z. The output (Y) and associated *H*(*Y*|*X, Z, U*) is interpreted as the information contained in Y but not in X, Z, and U. The LCF (Z) and associated *H*(*Z*|*X, Y, U*) is interpreted as the information contained in Z but not in X, Y, and U. The RF (X) and associated *H*(*X*|*Y, Z, U*) is interpreted as the information contained in X but not in Y, Z, and U.

A Venn diagram of the information theoretic measures for distinctive integrated input variables is depicted in Figure 6, where RF, LCF, and UCF are represented by the green, orange, and grayish pink ellipses, respectively. The output (Y) is represented by the blue ellipse. In information processing equations, the output is denoted by the random variable Y, whereas RF, LCF, and UCF are represented by X, Z, and U, respectively.

The mutual information shared between random variables X (RF) and Y (output) can be written as (Kay and Phillips, 2011):

(8) $$\begin{array}{l} I\left(X;Y\right)=H\left(X\right)-H\left(X|Y\right) \end{array}$$

Where, H(X) is the Shannon entropy associated with the distribution of X and *H*(*X*|*Y*) is the Shannon entropy associated with the conditional distribution of X given Y. It is defined as the information contained in X but not in Y (Kay and Phillips, 2011). It is assumed that the mutual information is always non-negative when random variables are stochastically independent (Kay and Phillips, 2011). Since we are dealing with four random variables, the conditional mutual information can be written as:

(9) $$\begin{array}{l} I\left(X;Y|Z,U\right)=H\left(Y|Z,U\right)-H\left(Y|X,Z,U\right) \end{array}$$

This is the conditional mutual information shared between X and Y, having observed Z and U. It is defined as the information shared between X and Y but not shared with Z and U.

The four-way mutual information shared among four random variables X, Y, Z, and U can be defined as:

(13) $$\begin{array}{l} I\left(X;Y;Z;U\right)=I\left(X;Y\right)-I\left(X;Y|Z,U\right)\\ \;=I\left(X;Z\right)-I\left(X;Z|Y,U\right)=\\ \;I\left(X;U\right)-I\left(X;U|Y,Z\right)=I\left(Y;Z\right)-I\left(Y;Z|X,U\right)\\ \;=I\left(Y;U\right)-I\left(Y;U|X,Z\right) \end{array}$$

If the four-way mutual information is positive, Shannon entropy associated with the distribution of Y can be defined as (Kay and Phillips, 2011):

(14) $$\begin{array}{l} H\left(Y\right)=I\left(Y;X;Z;U\right)+I\left(Y;X|Z,U\right)+I\left(Y;Z|X,U\right)\\ +I\left(Y;U|X,Z\right)+H\left(Y;X|Z,U\right) \end{array}$$

In case the random variables are discrete, the integrals are replaced by summations, and the probability mass function can be written as (Kay and Phillips, 2011):

(15) $$\begin{array}{lll} H\left(Y\right) & = & -\int p\left(y\right)\log p\left(y\right)dy \end{array}$$

(16) $$\begin{array}{lll} H\left(Y|X\right) & = & -\int \int p\left(y|x\right)\log p\left(y|x\right)p\left(x\right)dydx \end{array}$$

(17) $$\begin{array}{l} H\left(Y|X,Z\right)=-\int \int \int p\left(y|x,z\right)\\ \;\log p\left(y|x,z\right)p\left(x,z\right)dydxdz \end{array}$$

(18) $$\begin{array}{l} H\left(Y|X,Z,U\right)=-\int \int \int \int p\left(y|x,z,u\right)\\ \;\log p\left(y|x,z,u\right)p\left(x,z,u\right)dydxdzdu \end{array}$$

The objective function to be maximized can be defined as:

(19) $$\begin{array}{l} F=\phi_{0}I\left(Y;X;Z;U\right)+\phi_{1}I\left(Y;X|Z,U\right)\\ \;+\phi_{2}I\left(Y;Z|X,U\right)+\phi_{3}I\left(Y;U|X,Z\right)\\ +\phi_{4}H\left(Y;X|Z,U\right) \end{array}$$

*I*(*Y*; *X*|*Z, U*) is the information that the output shares with the RF (X) and is not contained in the LCF and UCF units. *I*(*Y*; *Z*|*X, U*) is the information that the output shares with the LCF and not contained in the RF and UCF units. *I*(*Y*; *U*|*X, Z*) is the information that the output shares with the UCF and not contained in the RF and LCF units.

The values of ϕ′*s* are tunable within the range [−1, 1]. Different ϕ values allow investigating specific mutual/shared information, such that:

$$f\left(x\right)=\{\begin{array}{l} F=I(Y;X),\text{ if }\phi_{1}=1,\phi_{2}=\phi_{3}=\phi_{4}=0\\ F=I(Y;Z),\text{ if }\phi_{2}=1,\phi_{1}=\phi_{3}=\phi_{4}=0\\ F=I(Y;U),\text{ if }\phi_{3}=1,\phi_{1}=\phi_{2}=\phi_{4}=0\\ I(Y;X;Z;U),\text{ otherwise} \end{array}$$

## 4. Case Study: Human Behavioral Modeling/AV Speech Processing

Human speech recognition in a noisy environment is known to be dependent upon both aural and visual cues, which are combined by sophisticated multi-level integration strategies to improve intelligibility (Adeel et al., 2019b). The correlation between the visible properties of articulatory organs (e.g., lips, teeth, tongue) and speech reception has been previously shown in numerous behavioral studies (Sumby and Pollack, 1954; McGurk and MacDonald, 1976; Summerfield, 1979; Patterson and Werker, 2003). Therefore, clear visibility of some articulatory organs could be effectively utilized to extract a clean speech signal out of a noisy audio signal. The proposed CMI theory is evaluated using human AV speech modeling. The developed AV models are illustrated in section 1 and Figure 2.

### 4.1. Audio-Visual Corpus and Feature Extraction

For contextual AV speech modeling, the AV ChiME3 corpus is developed by mixing the clean Grid videos (Cooke et al., 2006) with the ChiME3 noises (Barker et al., 2015) [cafe, street junction, public transport (bus), pedestrian area] for signal-to-noise ratio (SNRs) ranging from −12 to 12 dB (Adeel et al., 2019b). The pre-processing includes sentence alignment and incorporation of prior visual frames. Sentence alignment is performed to remove the silence time from the video and prevent the model from learning redundant or insignificant information. Prior multiple visual frames are used to incorporate temporal information to improve mapping between visual and audio features. The Grid corpus comprises 34 speakers, each speaker reciting 1,000 sentences. Out of 34 speakers, a subset of 5 speakers is selected (two white females, two white males, and one black male) with a total of 900 command sentences each. The subset fairly ensures the speaker independence criteria (Adeel et al., 2019b). A summary of the acquired visual dataset is presented in Tables 1, 2, where the full and aligned sentences, total number of sentences, used sentences, and removed sentences are clearly defined (Adeel et al., 2019b).

**Table 1:** Used grid corpus sentences (Adeel et al., 2019b).

			**Full sentences**		**Aligned sentences**	
**Speaker ID**	**Grid ID**	**No. of sentences**	**Removed**	**Used**	**Removed**	**Used**
Speaker 1	S1	1,000	11	989	11	989
Speaker 2	S15	1,000	164	836	164	836
Speaker 3	S26	1,000	16	984	71	929
Speaker 4	S6	1,000	9	991	9	991
Speaker 5	S7	1,000	11	989	11	989

**Table 2:** Summary of the train, test, and validation sentences (Adeel et al., 2019b).

**Speakers**	**Train**	**Validation**	**Test**	**Total**
1	692	99	198	989
2	585	84	167	836
3	650	93	186	929
4	693	99	199	991
5	692	99	198	989
All	3,312	474	948	4,734

For audio features, log filter-bank (FB) vectors are used. The input audio signal is sampled at 50 kHz and segmented into *N* 16 ms frames with 800 samples per frame and 62.5% increment rate. Afterwards, a hamming window and Fourier transformation is applied to produce the 2,048-bin power spectrum. Finally, a 23-dimensional log-FB is applied, followed by the logarithmic compression to produce the 23-D log-FB signal (Adeel et al., 2019b).

The visual features are extracted from the Grid Corpus videos recorded at 25 fps using a 2D-DCT based standard and a widely used visual feature extraction method. Firstly, the video files are processed to extract a sequence of individual frames. Secondly, a Viola-Jones lip detector (Viola and Jones, 2001) is used to identify the lip-region by defining the Region-of-Interest (ROI) in terms of a bounding box. Object detection is performed using Haar feature-based cascade classifiers. The method is based on machine learning where cascade function is trained with positive and negative images. Finally, the object tracker (Ross et al., 2008) is used to track the lip regions across the sequence of frames. The visual extraction procedure produced a set of corner points for each frame, where lip regions are then extracted by cropping the raw image. In addition, to ensure good lip tracking, each sentence is manually validated by inspecting a few frames from each sentence. The aim of manual validation is to delete those sentences in which lip regions are not correctly identified (Abel et al., 2016; Adeel et al., 2019b).

## 5. Experiments

Signal processing in the cerebral cortex is expected to comprise a common multipurpose algorithm that produces widely distributed but coherent and relevant activity patterns (Kay and Phillips, 2011). The coherent infomax exhibits specification of such algorithm. According to the theory of coherent infomax, local processors are able to combine reliable signal coding because of the existence of two classes of synaptic connections: driving connections (RF) and contextual connections (CF). The authors in Kay and Phillips (2011) made the biological relevance of this theory and showed that the coherent infomax is consistent with a particular Bayesian interpretation for the contextual guidance of learning and processing. However, this theory was evaluated using a simple edge detection problem to demonstrate the role of contextual modulation in improving feature detection with noisy inputs (Kay et al., 1998). The authors showed that how surrounding regions in different parallel streams (via contextual modulation) helped detecting the edges within any particular region and played a modulatory role in combating noisy input. More details including different properties of the coherent infomax are comprehensively presented in Kay and Phillips (1997), Kay et al. (1998), Phillips (2001), Phillips and Silverstein (2013), Kay et al. (2017), and Phillips et al. (2018).

**Figure 7:**
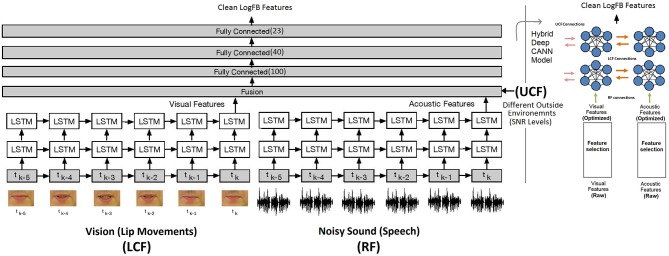
State-of-the-art network level AV deep LSTM model with 1000 LSTM and 162 MLP cells (noisy audio to clean audio mapping): CMI at the network level (Adeel et al., 2019a). The figure on the right depicts the hybrid deep LSTM-CANN model.

**Figure 8:**
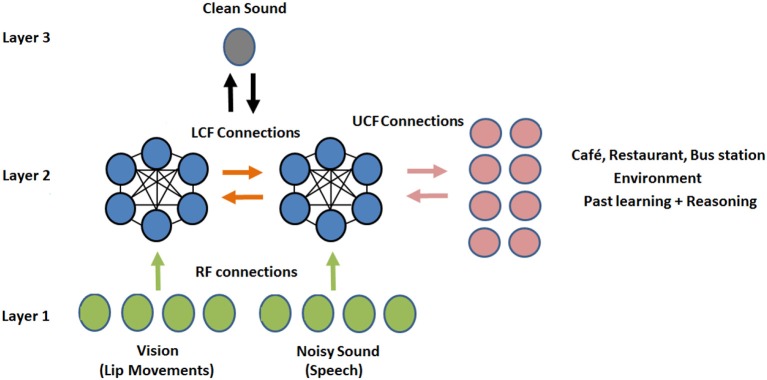
Proposed novel shallow CANN: CMI at the neural level. Three streams, three layered multisensory multiunit network of several similar CANs (where the CAN in one stream is connected to all other CANs in the neighboring streams). The CANN has 12 CANs, 4 RF neurons, 4 LCF neurons, 8 UCF neurons, and 1 output neuron, in total 29 neurons.

Going beyond a simple edge detection problem, in this subsection, we demonstrate how parallel streams constituting visual information play a modulatory role to disambiguate the noisy speech signal. For this, a hybrid deep LSTM and CANN models are developed for network and neural level multisensory integrations, shown in Figure 7 (Adeel et al., 2019a) and Figure 8, respectively. In Figure 7, the model on the right depicts the hybrid deep LSTM-CANN, which is a part of our ongoing work. Both LSTM and CANN models are trained with the AV ChiME3 dataset for SNRs ranging form −12 to 12 dB. Noisy audio and visual features of time instance *t*_*k*_, *t*_*k*−1_, …, *t*_*k*−5_ are feeded into LSTM and CANN models. The aim is to map noisy audio to clean audio features. The first LSTM layer has 250 cells, which encoded the input and passed its hidden state to the second LSTM layer, which has 300 cells. Finally, the optimized latent features from both LSTM models are fused using two dense layers. Specifically, the optimal features extracted from each LSTM network are concatenated into a single vector. The concatenated vector is then feeded into a fully connected multilayered perceptron (MLP) network. The MLP network comprises three layers with 100 and 40 ReLU neurons in the first two layers and 22 linear neurons in the last layer. The UCF is integrated in the second last layer. The training procedure for CANN is comprehensively presented in section 5.3.1. Both deep LSTM and CANN architectures are trained with the objective to minimize the mean squared error (MSE) between the predicted and the actual clean audio features. The MSE (20) between the estimated audio logFB features and clean audio features is minimized using the stochastic gradient decent algorithm and the RMSProp optimizer. RMSprop is an adaptive learning rate optimizer which divides the learning rate by the moving average of the magnitudes of recent gradients to make learning more efficient. Moreover, to reduce overfitting, dropout (0.20) was applied after every LSTM layer. The MSE cost function *C*(*a*_*estimated*_, *a*_*clean*_) can be written as (Adeel et al., 2019b):

(20) $$C(a_{\text{estimated}},a_{\text{clean}})=\sum_{i=1}^{n}0.5{\left(a_{\text{estimated}}(i)-a_{\text{clean}}(i)\right)}^{2}$$

where *a*_*estimated*_ and *a*_*clean*_ are the estimated and clean audio features, respectively.

### 5.1. Single Stream: RF Only

**Figure 9:**
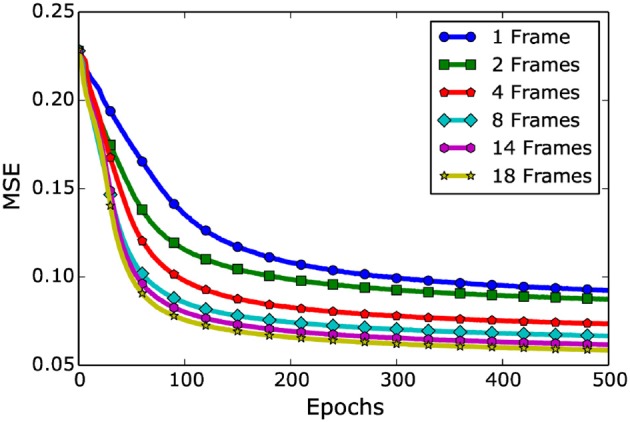
Single stream (RF only): Network level visual only (lip-reading driven) deep learning performance. The figure presents an overall behavior of an LSTM model when different number of visual frames are added. Please note the improvement in learning upon prior visual frames integration (Adeel et al., 2019b).

The deep LSTM model is trained only with visual cues (RF only) considering multiple prior frames (ranging from 1 visual frame to 18 prior visual frames). The simulation results are shown in Figure 9 (Adeel et al., 2019b). The training is performed with six different aligned datasets (i.e., 1, 2, 4, 8, 14, and 18 prior visual frames). It can be seen that by moving from 1 visual frame to 18 visual frames, a significant performance improvement could be achieved. The LSTM model with 1 visual frame achieved the MSE of 0.092, whereas with 18 visual frames, the model achieved the least MSE of 0.058. The LSTM model exploited the temporal information effectively and showed consistent reduction in MSE. This is mainly because of its inherent recurrent architectural property and the ability of retaining state over longer time spans using cell gates.

### 5.2. Parallel Multistreams: RF + LCF

**Figure 10:**
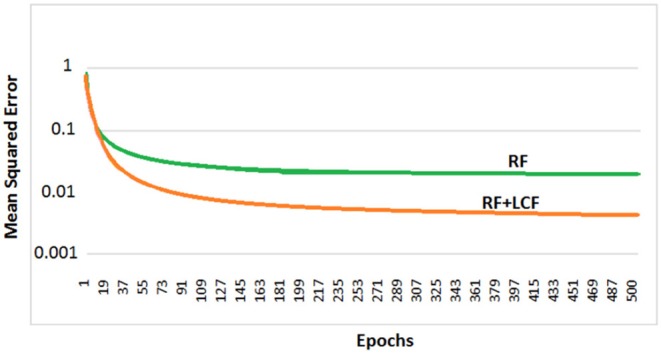
Parallel multistreams (RF + LCF): learning and processing results. It is to be noted that the integration of LCF significantly improved learning.

**Figure 11:**
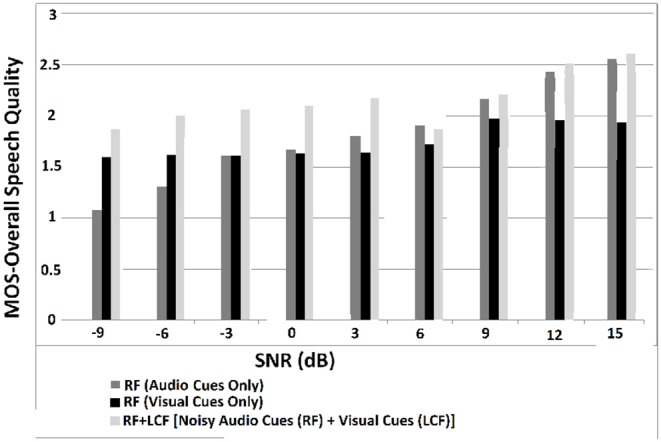
MOS for overall speech perception—these results are obtained through subjective listening tests, conducted in terms of MOS with self-reported normal-hearing listeners. The listeners were presented with a single stimulus (i.e., enhanced speech only) and were asked to rate the re-constructed speech on a scale of 1–5. The five rating choices were: (5) Excellent (when the listener feels an unnoticeable difference compared to the target clean speech), (4) Good (perceptible but not annoying), (3) Fair (slightly annoying), (2) Poor (annoying), and (1) Bad (very annoying).

**Figure 12:**
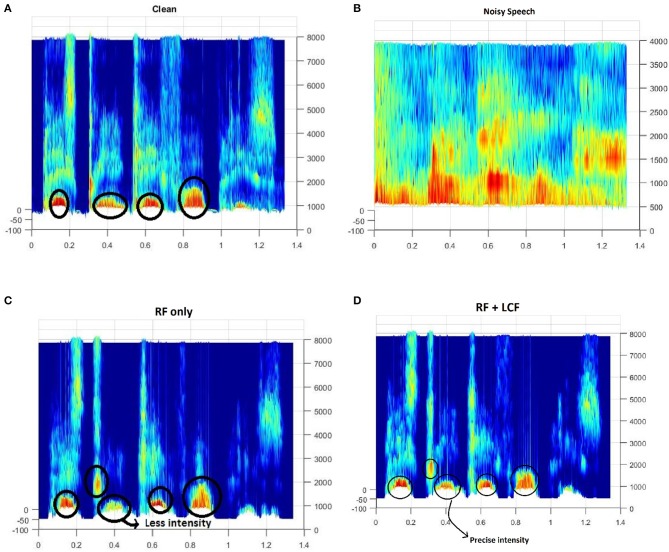
Spectrogram of a randomly selected utterance of −9dB SNR from AV ChiME3 corpus [X-axis: Time; Y-axis: Frequency (Hz)]: **(A)** Clean, **(B)** Noisy, **(C)** RF-only enhanced speech, **(D)** RF + LCF enhanced speech. Note that RF + LCF model recovered some of the frequency components better than RF-only at low SNR.

In this experiment, the deep LSTM model is feeded with noisy audio cues (as RF) and visual cues (as LCF). The training results of AV model (RF + LCF) are depicted in Figure 10, where the improvement in learning and processing due to LCF integration is evident (Adeel et al., 2019a). The speech perception results in terms of speech quality are shown in Figure 11. The used speech enhancement framework is out of the scope of this paper and is comprehensively presented in Adeel et al. (2019b). It can be seen that at high level of background noise (e.g., busy restaurant), visual-only cues are outperforming audio-only cues. In contrast, at low level of background noise (high SNR), audio-only cues are outperforming visual-only cues. It shows that visual cues are fairly less effective for speech enhancement at low or zero background noise which is analogous to human audio-visual speech processing. However, AV (RF + LCF) model outperforms both audio-only and visual-only models in all situations (at both low and high SNRs). The AV model is leveraging the complementary strengths of both audio and visual cues. At high background noise, LCF is acting as a modulatory signal and helping the model to disambiguate the noisy audio speech signal. At low level of background noise, the role of LCF starts decreasing [eventually reaches to Null (hypothetically)—ceiling effect]. This phenomenon is more clear in Figure 12, where the spectrogram of a randomly selected utterance of −9 dB SNR is depicted. However, for in-depth and neural level analysis, the CANN is modeled and trained in section 5.3.1 to enable better quantification of this amplification and suppression process.

### 5.3. Beyond Coherent Infomax: RF + LCF + UCF

**Figure 13:**
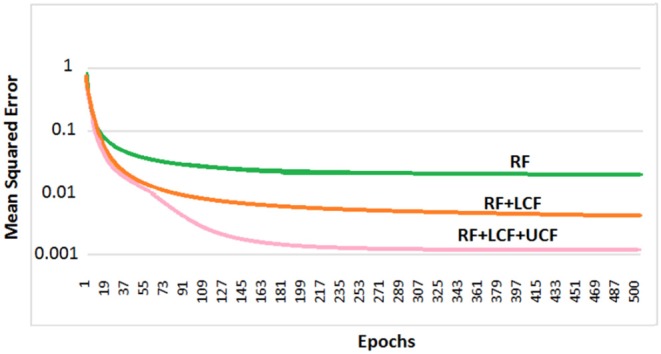
Deep LSTM learning and processing results: it is to be noted that RF + LCF + UCF model outperforms both RF-only and RF + LCF models.

So far, it is seen how LCF could play a modulatory or null role upon changing the context (outside environment). However, contextual identification and transition (from one context to another) are two difficult problems. Given any desired human behavior to be modeled, a set of appropriate contexts associated with the anticipated behaviors and actions could be identified and grouped together to develop a computationally efficient model (given a broader understanding of the task in hand) (Gonzalez et al., 2008). In this subsection, the deep LSTM model is trained with three distinctive multimodal multistreams: lip movements as LCF, noisy speech as RF, and the outside environment/anticipated behavior as UCF. For contextual information (UCF integration), five dynamic real-world commercially-motivated scenarios are considered: cafe, restaurant, public transport, pedestrian area, and home. Please note that a specific SNR range defines a particular environment (UCF), represented by a unique pattern. The training results are presented in Figure 13 where a significant improvement in learning is evident. Hence, given a broader understanding of the task in hand (acquired through incoming sensory signals (having a high correlation to the external world), specific situation, and associated anticipated behavior), an enhanced learning and optimized decision making could be achieved.

#### 5.3.1. CANN: RF + LCF + UCF

So far, the end-to-end multimodal deep learning models have demonstrated CMI at the network level. However, the underlying neural processing in deep learning models is elusive and it is difficult to analyze the precise information processing. For example, in case of deep LSTM driven AV processing, it is difficult to quantify the selective amplification or suppression of multisensory AV information at different levels. To address these problems, the proposed novel AV-CANN model (shown in Figure 8) is trained using AV ChiME3 corpus. For training, the deep problem was transformed into a shallow problem. Specifically, the evaluated shallow CANN model predicts one coefficient at a time (i.e., coefficient by coefficient prediction). The data samples include 2D-logFB (speech features) and 2D-DCT (visual features) coefficients for 1,000 utterances from Grid and ChiME3 Corpora (Speaker 1 of the Grid). The number of clean logFB audio features are 22 × 205,712. The combined noisy logFB audio features are 22 × 205,712 (for −12, −9, −6, −3, 0, 3, 6, 9, and 12 dB SNRs). Similarly, the DCT visual features are 25 × 205,712 in total.

In AV CANN model, the filter bank (audio cues) and DCT (visual cues) coefficients are represented as signals coming from the outside world according to Poisson arrival streams of rates (Λ_*x*_, λ_*x*_). These inputs are converted into average rate of positive and negative signals, given by Equations (9) and (10). Specifically, a set of successive inputs is denoted as X = (*x*^(1)^…., *x*^(*K*)^), Where, x(k) = ($\Lambda_{x}^{\left(k\right)}$, $\Lambda_{x}^{\left(k\right)}$) are pairs of excitation and inhibition signals entering each neuron from the outside world.

**Figure 14:**
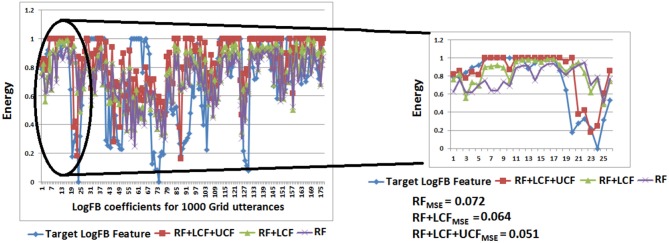
CANN learning and processing results: clean audio features prediction with A-only (RF), AV (RF + LCF), and AV with UCF models. It is to be noted that RF + LCF + UCF model outperforms both RF-only and RF + LCF models.

Figure 14 depicts the prediction of clean logFB coefficients, where it can be seen that RF + LCF + UCF model outperformed both RF + LCF and RF-only models, achieving MSE of 0.051, 0.064, and 0.072, respectively. It is also worth mentioning that the shallow CANN with only 29 spiking neurons performed comparably to deep LSTM unimodal network (RF-only). In conjunction with the coherent infomax theory, the enhanced learning in CANN is due to a widely distributed and shared activity pattern. The CANN discovered and exploited the associative relations between the features extracted within each of the RF, LCF, and UCF streams.

## 6. Discussion and Conclusions

It is worth mentioning that the author has not claimed to know the origin of consciousness, instead, proposed a theory on its possible function in multisensory integration. A two-compartment neuron with distinct somatic (RF) and apical (CF) zones of integration is well-established (Larkum et al., 2009; Kay and Phillips, 2011; Larkum, 2013; Larkum and Phillips, 2016) and supported for effective learning in deep networks (Lillicrap et al., 2020). However, the apical input (CF), coming from the feedback and lateral connections, is far more diverse with far greater implications for ongoing learning and processing in the brain. CMI theory emphasizes the importance of understanding and defining the roles of different kinds of contexts in pyramidal cells. Thus, it puts forward the idea of dissecting CF into LCF and UCF, to better understand the amplification and suppression of relevant and irrelevant signals, with respect to different external environments and anticipated behaviors.

Preliminary results shed light on selective amplification/attenuation of AV signals. It is shown that in different environmental conditions (represented as UCF), roles of audio and visual cues change, e.g., in high-level of background noise, visual cues (as LCF) modulate the noisy audio cues (RF), whereas, in low-level of background noise, LCF becomes relatively less effective, with no role (hypothetically) in zero background noise. Furthermore, in terms of enhanced learning and processing, the parallel three-stream (RF + LCF + UCF) deep neural network model out performs the parallel two-stream (RF + LCF) and single-stream (RF-only) models. Similar results are obtained with a shallow contextually-adaptive neural network (CANN), which also enables quantification of multiway mutual/shared information at the neural level. The integration of RF, LCF, and UCF guides learning and processing while enables the network to explore and exploit the associative relations between the features extracted within different fields for optimized decision making.

These findings suggest that the pyramidal cell, in addition to the classical excitatory and inhibitory signals, receives the LCF and UCF inputs. The UCF (as a steering force or tuner) helps pyramidal cells in precisely selecting the relevant or useful feedforward signals from overwhelming available information and deciding whether to amplify/suppress their transmission e.g., which information is worth paying more attention to? This is called conditional amplification/suppression (with respect to the outside world) as opposed to unconditional excitatory and inhibitory activity in existing DNNs.

The distinctive role of UCF (as a tuner) quite strongly implicates that it is closely related to consciousness (Bachmann and Anthony, 2014; Phillips et al., 2016). Overall, the proposed CMI theory improves our understanding of the mechanisms responsible to produce coherent thoughts, percepts, and actions, which are well-adapted to different situations and long-term goals. The distinction between different contextual fields (LCF and UCF) is certainly a move in the right direction. However, the presented basic mathematical model and results should be taken with care. The CMI neural model needs to be significantly improved by incorporating the observed network behavior, which conditionally amplifies/suppresses the RF-LCF signals with respect to different external environments.

## 7. Future Research Directions

Future work aims to quantify the suppression and attenuation of multisensory signals in terms of four basic arithmetic operators (addition, subtraction, multiplication and division) and their various forms (Kay et al., 2017). We will analyze how the information in CANN is decomposed into components unique to each other having multiway mutual/shared information. The ongoing and future work also includes studying the application of CMI theory to a range of real-world problems, including: (i) computational modeling of AV processing in Alzheimer's and Parkinson's diseases, and schizophrenia (Phillips et al., 2015, 2016), (ii) natural human-robot interactions, (iii) low-power neuromorphic chips, (iv) brain-computer interface, and (v) neurofinance. Furthermore, several other areas within psychology and neuroscience could potentially benefit from the proposed theory (e.g., Héericé et al., 2016; Karim et al., 2017). Defense Advanced Research Projects Agency (DARPA) recently announced a USD 2 billion campaign to develop the next wave of artificial intelligence (AI) technologies (Szu et al., 2019). Specifically, DARPA seeks contextual reasoning in AI systems to create more collaborative and trusting partnerships between humans and machines. In this context, the proposed theory represents a step change.

## Data Availability Statement

The datasets generated for this study are available on request to the corresponding author.

## Author Contributions

The author confirms being the sole contributor of this work and has approved it for publication.

## Conflict of Interest

The author declares that the research was conducted in the absence of any commercial or financial relationships that could be construed as a potential conflict of interest.

## Footnotes

^1^Effective connectivity is the ability of neuronal groups to causally affect other neuronal groups within a system.
